# Case report: The intrauterine suture surgery—an original method in the treatment of old uterine false passage

**DOI:** 10.3389/fsurg.2023.1193961

**Published:** 2023-07-28

**Authors:** Xiaolei Song, Re Na, Nianghai Peng, Wenming Cao, Yan Ke, Ping Liu, Chunlin Chen

**Affiliations:** ^1^ Department of Gynecology, Shenzhen Hospital of Integrated Traditional Chinese and Western Medicine, Shenzhen, China; ^2^ Department of Gynecology, Southern Hospital of Southern Medical University, Guangdong, China

**Keywords:** new technique of intrauterine suture, old uterine false passage, intrauterine adhesions, conservative treatment failure, abnormal uterine bleeding

## Abstract

**Objective:**

To introduce an effective approach using the hysteroscopy system for patients with old uterine false passage after a failed conservative treatment.

**Materials and methods:**

This study presents the case of a 34-year-old woman who was treated in the Department of Gynecology of Shenzhen Integrated Traditional Chinese and Western Medicine Hospital in 2018 with the complaint of “menstrual volumereduction for 2 years after abortion.” A hysteroscopy was performed to make a clear diagnosis: (1) uterine cavity adhesion and (2) old uterine false passage. After the separation of adhesions, the patient was treated with estradiol and progesterone in sequence (estradiol valerate 3 mg, b.i.d., oral for 21 days; and dydrogesterone tablets 10 mg, b.i.d., oral for the second half of the cycle) for 3 months. After the review of the hysteroscopy results, it was found that there was no improvement in the old false passage; therefore, a suture and knotting surgery under hysteroscopy was performed to treat the old false passage in the uterus within 10 min, and the intraoperative blood loss was 2 ml. The patient was discharged 24 h postoperatively without any adverse perioperative complications.

**Results:**

Two months after the operation, the review of the hysteroscopy results showed that the old false passage in the uterus disappeared. After the 6-month follow-up, the menstrual volume increased compared with the previous one, close to the normal menstrual volume, and the patient experienced no pain and menstrual discomfort. The patient was lost to follow-up and was contacted again in 2022. It was found out that in 2019, she was pregnant with a baby boy who is now 3 years old and healthy.

**Conclusion:**

The intrauterine suture surgery presents a clear visual ﬁeld to old uterine false passage after a failed conservative treatment. In patients with old uterine false passage suffering from reduced fertility, the intrauterine suture surgery can be a minimally invasive and effective alternative if the conservative treatment for old uterine false passage failed.

## 1. Introduction

A 38-year-old woman was admitted to the Department of Gynecology, Shenzhen Hospital of Integrated Traditional Chinese and Western Medicine, with the complaint of “menstrual volume reduction for 2 years after abortion.” The patient had regular menstruation for 5/28–32 days. The menstrual volume decreased by one-half after abortion 6 years ago, and 2–3 daily sanitary pads (one-third soaked) were used daily for 4 days. The menstrual period can extend to 6–7 days without dysmenorrhea. The last menstruation was on 12 March 2018. Since the onset of the disease, the patient did not experience chills, fever, or in low spirit; had no significant change in diet and sleep, had normal urine and stool characteristics, and no significant increase or decrease in body weight.

Menstrual history: the age at menarche was 15 years old, menstrual cycle 28–32 days, menstrual period 6–7 days, moderate menstrual volume, menstrual volume decreased by one-half in the past 2 years, menstrual period extended to 6–7 days, no dysmenorrhea, last menstruation: 12 March 2018, and leukorrhea is normal and homogeneous.

History of marriage and childbirth: married, healthy spouse, 2-0-8-2, cesarean section two times, abortion four times, drug abortion four times, the existing two; there were three previous history of intrauterine device (IUD) insertion (including two pregnancies with IUD). The last pregnancy: 6 February 2016, the outcome was 6+ weeks of pregnancy induced abortion + ring.

Laboratory examination: transvaginal color Doppler ultrasound: there was no obvious abnormal sound image in the uterus and bilateral accessory area.

Diagnosis: intrauterine adhesions

**Figure 1:**
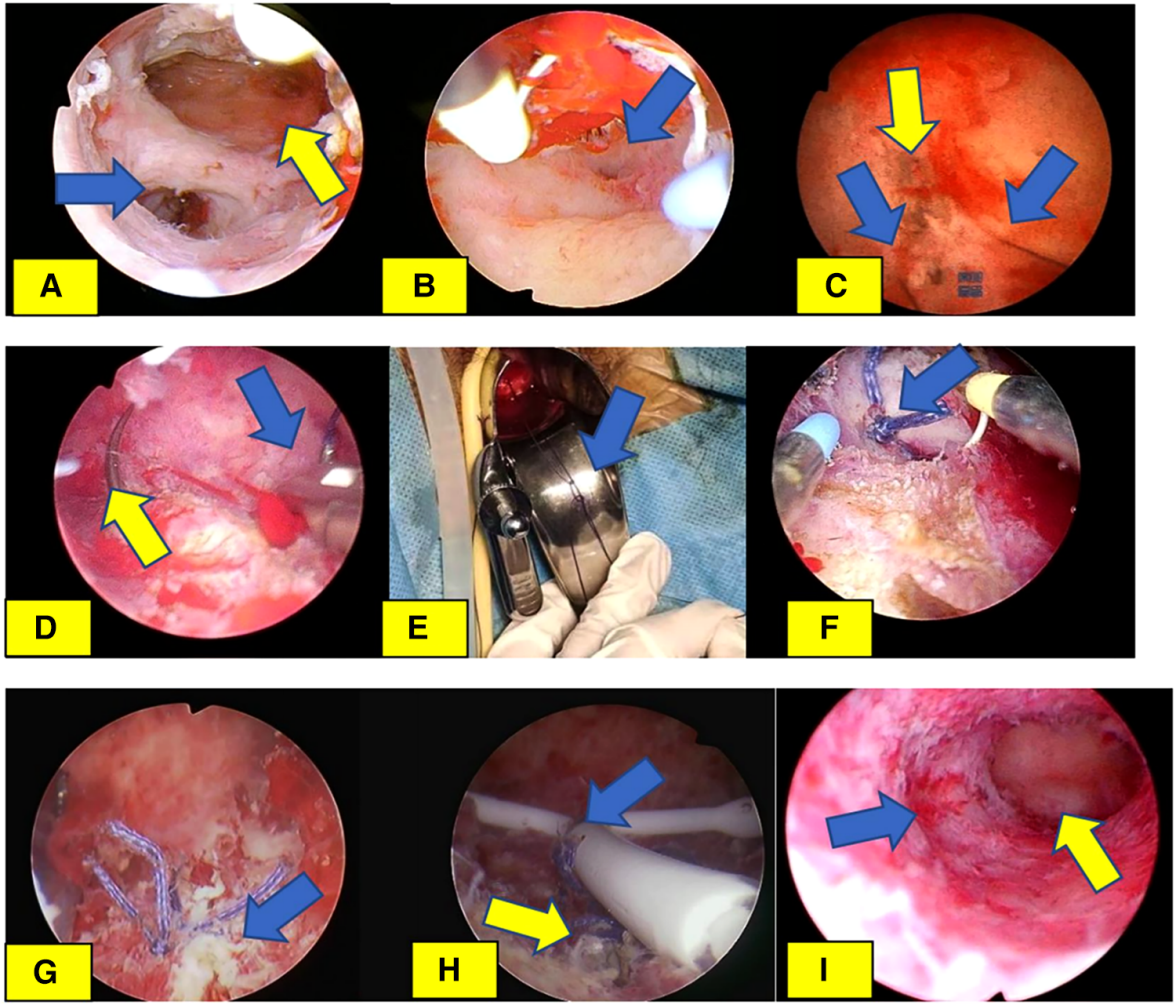
Hysteroscopic suture knotting and intrauterine device suture fixation of uterine old false passage. (**A**) In the first hysteroscopy, the blue arrow refers to the old false passage, and the yellow arrow refers to the uterine cavity. (**B**) The blue arrow is the opening of the fallopian tube, which is confirmed as the uterine cavity again. (**C**) Suture knotting of intrauterine lesions. The blue arrow refers to the 2-0 absorbable suture on both sides of the cut old false passage, and the yellow arrow refers to the cut strip old false passage. (**D**) The suture needle of the suture of intrauterine disease penetrates the details of the intrauterine tissue. The blue arrow refers to the tail of the needle and the yellow arrow refers to the tip of the needle. (**E**) Knotting details of sutures for intrauterine diseases (double slip knots of what we call butterfly bows, we named it Song's knot), shape of knots indicated by blue arrows. (**F**) Confirm the details of the bow lock tissue under hysteroscopy (shown in blue arrows). (**G**) Old uterine false passage after suture knot under hysteroscopy (shown in blue arrows). (**H**) The state after suture knotting and suture fixation of the old false passage. The yellow arrow refers to the state after suture knotting of the false passage, and the blue arrow refers to the state after suture fixation of the LIN-US band. (**I**) After 2 months of follow-up, the Mirena ring was removed and compared with (**A**). The blue arrow indicated the closure of the old false passage, and the yellow arrow indicated the uterine cavity.

Diagnosis and treatment: the patient was examined after admission, and hysteroscopy was performed after the exclusion of surgical contraindications to confirm the diagnosis: (1) intrauterine adhesions and (2) old uterine false passage (Figures 1A,B). After the separation of adhesions, sequential treatment of estrogen and progesterone (estradiol valerate 3 mg, b.i.d., oral for 21 days; and dydrogesterone tablets 10 mg, b.i.d., oral for the second half of the cycle) was given for 3 months. After the review of the hysteroscopy results, it was found that there was no improvement in the old false passage. Therefore, a hysteroscopic suture knotting was performed to treat the old false passage in the uterus, and the intrauterine device was sutured and fixed in the uterine cavity (Figures 1C–H). Two months after the operation, the patient had fertility needs due to marital issues. The removal of the intrauterine device was required, and the hysteroscopy results showed that the old false passage in the uterus disappeared (Figure 1I). There was no discomfort experienced such as dysmenorrhea. Eventually, the patient was lost to follow-up. In 2022, she was contacted again and revealed that she had given birth to a baby boy at full term in 2019, who is now 3 years old and healthy.

## 2. Discussion

### 2.1. Introduction to intrauterine suturing under hysteroscopy

Hysteroscopic intrauterine suture is a minimally invasive procedure, which is the continuation of the basic operations of surgery: incision, suture, knot tying, and thread cutting in the uterine cavity. It is applicable to cases where a repair of the endometrium or uterine wall is required or to fix an intrauterine device. This surgery is guided by a hysteroscope to perform incisions, sutures, knots, and thread cutting operations inside the uterus, reducing damage to surrounding tissues and bleeding. The recovery time is also faster than the traditional surgery, reducing or avoiding the possibility of the intrauterine device falling off, moving in position, and rotating. It also allows some surgeries with stages that need to be completed in one go, saving medical resources and patient time and money costs. This type of surgery is usually performed by experienced gynecologists (1).

### 2.2. Overview of uterine prostheses

Uterine wall prosthesis is a difficult issue in the diagnosis and treatment of gynecological intrauterine diseases. The incidence of uterine wall prosthesis reported in literature is 0.12%–3.33% (2–4), which is not uncommon. However, there is currently limited research on its management and no unified treatment plan available. The definition of uterine wall prosthesis refers to the damage of the uterine muscle wall from the uterine cavity toward the uterine serosa layer, which can also be referred to as incomplete uterine perforation or poor healing after uterine perforation. If not diagnosed and treated in time, it will lead to a series of complications, such as abnormal uterine bleeding, uterine rupture during pregnancy, infection, sinus formation, and also increase the risk of uterine perforation for the subsequent hysteroscopic surgeons. The formation of uterine prostheses is often related to difficult uterine cavity procedures, such as uterine curvature, cervical atrophy, cervical adhesions, and uterine cavity adhesions. The clear diagnosis is mostly found during hysteroscopic surgery. As for its treatment, in the past, it was often used to promote uterine contraction, prevent infection, promote the healing of the false passage, or perform sequential treatment with estrogen and progestogen (5–8), looking forward to its self-healing. Some scholars cooperated with hysteroscopic positioning, and closed the false passage with the help of suture surgery under the abdominal endoscope. For the false passage with long formation time and cicatricial non-union, the position, size, and depth of the false passage should be clearly described, and the succeeding operation in the uterine cavity should be cautiously performed to avoid perforation of the false passage. Such treatment is ineffective for the patients who have fertility requirements or who are forbidden to take estrogen and progestogen drugs, or it may delay the delivery date of the patient. At the same time, the intake of exogenous estrogen and progestogen increases the medical cost of the patient. It also increases the risk of patients developing thrombosis, endometrial tumors, and breast tumors. At present, the operation team of the author innovatively uses intrauterine disease suture and knotting technique to treat old uterine false passage with positive outcome, avoiding the uncertainty of the traditional treatment of such patients and the risks and costs caused by large doses of oral estrogen and progestogen. In this operation, the surgical team uses ordinary hysteroscopy instruments to complete the intrauterine suture treatment of old uterine false passage without the use of special surgical instruments. The operation time is 7 min and 21 s, which is relatively short. Therefore, this surgery has a stronger public foundation and better learnability, inheritability, and transmissibility, which helps clinical professionals master the technique and provide services for human health. The novelty report from the Guangdong Provincial Medical Academic Exchange Center confirms that this surgical method is the first domestically and internationally performed surgery.

### 2.3. Key points and indications of intrauterine suturing for the treatment of uterine prostheses

The surgical team searched the literature, and found no report of hysteroscopic suture of uterine false passage. In this report, we first talked about the surgical instruments and sutures used. The author team used ordinary hysteroscopy instruments and selected a 3 mm needle holder for laparoscopy, which were inserted together into the uterine cavity through the cervix. Under the condition of uterine distention, we completed the suture and knotting of intrauterine diseases under hysteroscopy, and fixed the suture through the knotting method that we designed. The problem of suture operation was successfully solved. Previously, the surgical team had also attempted to complete the suture of uterine prostheses under hysteroscopy using a 10 mm hysteroscope and a 5 mm laparoscopic needle holder, but the difficulty was relatively greater than using a 3 mm needle holder. The suture used by the surgical team is a 2-0 absorbable suture with a needle curvature of 5/8C and a length of 26 mm. The suture is completed under hysteroscopy and then tied and fixed. For tying, we can use a knotter to complete the suture, but there is a problem of loose knots. The knotting technique mentioned in the article (we named it Song's knot) solves this problem. Although the surgical knots completed under this technique have the characteristics of loose knots, it can firmly lock the sutured tissue, solving the problem of loose knots. In addition, we should objectively see that this technology is a continuation of the basic surgical techniques under hysteroscopy, an innovation in thinking, and also provides patients with another option of treatment. At present, this technology has been applied to the following special patients: patients with endometrial myometrial defect who failed to receive or refused to receive conservative management, or patients with intrauterine bleeding that affected the surgical field of vision; suturing of the uterine submucosal fibroids after surgery; patients with excessive menstruation related to adenomyosis who undergo endometrial removal surgery and experience recurrent uterine bleeding 6 months or several years later; patients with thrombocytopenia and uterine bleeding caused by hematological diseases such as leukemia; patients with excessive menstruation related to adenomyosis who undergo other treatments and experience recurrent uterine bleeding several years later; patients with abnormal uterine cavity morphology and chromosomal abnormalities who do not require contraception; patients who have experienced detachment, rotation, or displacement of the intrauterine device after placement, or patients who have experienced multiple instances of intrauterine device detachment; patients with rotation and abnormal uterine bleeding after anchoring the intrauterine slow release system; and patients with abnormal uterine bleeding caused by other bleeding diseases (such as uremia dialysis patients, cerebral hemorrhage patients). The application of this technology will help decrease the surgical costs of the patient. The fastest completion time for intrauterine suture under hysteroscopy of the team is 1 min and 23 s, and it does not increase the incidence of surgical complications for patients. From the perspective of health economics, it saves medical and health resources, and retaining the uterus can save the expenses of the patient, as hysterectomy surgery requires more medical resources and costs including operating room use, anesthesia, beds, and medication. In addition, a certain recovery time and rehabilitation treatment are required after hysterectomy, which will also increase the expenses of the patient.

In short, as a new approach and continuation of basic surgical procedures in the uterine cavity, hysteroscopic intrauterine suture surgery demonstrates another way to manage intrauterine diseases and provides patients with more options of treatment.

## Data Availability

The original contributions presented in the study are included in the article, further inquiries can be directed to the corresponding authors.
